# Risk Factors for Severe Neutropenia following Intra-Arterial Chemotherapy for Intra-Ocular Retinoblastoma

**DOI:** 10.1371/journal.pone.0108692

**Published:** 2014-10-10

**Authors:** Ira J. Dunkel, Weiji Shi, Kim Salvaggio, Brian P. Marr, Scott E. Brodie, Y. Pierre Gobin, David H. Abramson

**Affiliations:** 1 Department of Pediatrics, Memorial Sloan-Kettering Cancer Center, New York, NY, United States of America; 2 Department of Pediatrics, New York Presbyterian Hospital Weill Cornell Medical College, New York, NY, United States of America; 3 Department of Epidemiology and Biostatistics, Memorial Sloan-Kettering Cancer Center, New York, NY, United States of America; 4 Department of Neurosurgery, New York Presbyterian Hospital Weill Cornell Medical College, New York, NY, United States of America; 5 Department of Surgery, Memorial Sloan-Kettering Cancer Center, New York, NY, United States of America; 6 Department of Ophthalmology, New York Presbyterian Hospital Weill Cornell Medical College, New York, NY, United States of America; 7 Department of Ophthalmology, Mount Sinai Medical Center, New York, NY, United States of America; National Cancer Institute, United States of America

## Abstract

**Purpose:**

Intra-arterial chemotherapy is a promising strategy for intra-ocular retinoblastoma. Neutropenia is the most commonly encountered systemic toxicity and in this study we aimed to determine the risk factors associated with the development of severe (≥grade 3) neutropenia.

**Methods:**

Retrospective review of 187 evaluable cycles of melphalan-containing intra-arterial chemotherapy from the first three cycles administered to 106 patients with intra-ocular retinoblastoma from May 2006 to June 2011. Cycles were considered to be evaluable if (1) blood count results were available in the 7 to 14 days post-treatment interval and (2) concurrent intravenous chemotherapy was not administered. Toxicity was assessed via the Common Terminology Criteria for Adverse Events version 4.0.

**Results:**

54 cycles (29%) were associated with grade 3 (n = 43) or grade 4 (n = 11) neutropenia. Multivariate stepwise logistic regression revealed that a higher melphalan dose (>0.40 mg/kg) was significantly associated with severe neutropenia during all 3 cycles (odds ratio during cycle one 4.11, 95% confidence interval 1.33–12.73, p = 0.01), but the addition of topotecan and/or carboplatin were not. Prior treatment with systemic chemotherapy was not associated with severe neutropenia risk in any analysis.

**Conclusions:**

Intra-arterial melphalan-based chemotherapy can cause severe neutropenia, especially when a dose of greater than 0.40 mg/kg is administered. Further study with a larger sample may be warranted.

## Introduction

Retinoblastoma is the most common primary ocular tumor of childhood. In the United States and other socio-economically advantaged parts of the world, the vast majority of patients have intra-ocular disease at diagnosis. During the past 15 to 20 years advanced intra-ocular disease has most often been treated with enucleation, but super selective intra-arterial chemotherapy appears to be a promising option [1]–[2]. It is generally well tolerated, but grade 3 and 4 neutropenia may occur. We performed this analysis to try to determine risk factors associated with the development of severe neutropenia and hypothesized that factors associated with development of grade 3 or 4 neutropenia would be (1) the melphalan dose, (2) administration of topotecan and/or carboplatin in addition to melphalan, and (3) prior treatment with systemic chemotherapy.

## Methods

### Ethics statement

The Memorial Sloan-Kettering Cancer Center's Institutional Review Board/Privacy Board approved this retrospective review of existing data, granting a waiver for this to be done without obtaining consent from the subjects or their parents/legal guardians. All protected health information was handled in accordance with institutional policies that did not require the information to be anonymized and de-identified prior to analysis.

### Patients

We retrospectively reviewed the first 106 consecutive patients with intra-ocular retinoblastoma treated with intra-arterial chemotherapy at our centers from May 2006 to June 2011.

Patients most frequently were treated with intra-arterial single-agent melphalan, but some cycles also included treatment with intra-arterial topotecan and/or carboplatin (Table 1). The number of agents to be used and the doses of chemotherapy administered were determined on a case by case basis. In general, patients received more than one agent if they had more severe disease or had been extensively pre-treated with intravenous chemotherapy and/or external beam radiation therapy, especially if we were treating the only remaining eye. In cycle 1, the dose was primarily determined by the patient's age. Patients 3 to 6 months of age generally received 2.5 mg of melphalan, 6 to 12 months of age, 3 mg of melphalan, 1 to 3 years of age, 4 mg of melphalan, and ≥3 years of age, 5 mg of melphalan [1]. In subsequent cycles we would consider increasing the dose(s) if the ophthalmic artery had large extra-ocular branches or if an inadequate response had been encountered without significant toxicity. We would consider decreasing the dose(s) if wedge flow was encountered or significant toxicity was encountered, such as an interval decrease in the eye's electroretinogram or an ocular inflammatory reaction. The median topotecan dose administered was 0.4 mg (range 0.2 to 2 mg) and the median carboplatin dose administered was 30 mg (range 25 to 80 mg).

**Table 1:** The number of patients treated per cycle with the various chemotherapy agents and the number of patients evaluable for analysis of neutropenia.

Cycle	Patient	M only	M + T	M+C	M+T+C	Inevaluable	Evaluable
1	106	74	21	1	10	33	73
2	100	67	25	1	7	36	64
3	86	53	24	1	8	36	50

M: melphalan; T: topotecan; C: carboplatin.

We asked the parents to have a complete blood count performed 7 to 10 days after each dose of intra-arterial chemotherapy. However, many families did not reside in the New York area and returned home after the treatment, and so compliance was variable. Intra-arterial chemotherapy cycles were considered to be evaluable if (1) blood count results were available in the 7 to 14 days post-treatment interval, and (2) concurrent intravenous chemotherapy was not administered. However, if a blood count was not available within the 7 to 14 day post-treatment window, but grade 3 or 4 neutropenia was documented earlier or later in the cycle, the cycle was considered evaluable. Toxicity was assessed via the Common Terminology Criteria for Adverse Events (CTCAE) version 4.0.

### Statistics

A binary melphalan dose is of interest in potential prediction. An optimal cut-off point of the dose was selected using Miller and Siegmund's minimum p-value approach and Altman, Lausen, Sauerbrei, and Schumacher's formula to adjust the minimum p-value selected from the systematic dependent multiple testing [3]–[4]. The highest and lowest 10% of the melphalan dose data were eliminated from the selection procedure. A univariate analysis of relationship between treatment factors and severe neutropenia (defined as grade 3 or 4 neutropenia) was performed using Chi-square, or Fisher's exact test. A multivariate analysis of the joint relationship was performed using a stepwise logistical regression method. Variables with p-value ≤0.20 on univariate analysis were candidates for the initial multivariate model. The statistical analysis was performed with the software SAS version 9.2 (SAS Institute Cary, NC) and r package ROCR (version 2.9.2). A p-value<0.05 was considered significant.

All analyses were based on evaluable cycles only. Grade 3 and 4 neutropenia were combined into the entity of severe neutropenia due to the small number of grade 4 neutropenia events (n = 5, 3, and 3 during cycles 1, 2 and 3, respectively).

## Results

### Melphalan dose and analysis of optimal cut-off point

A preliminary analysis showed that a higher melphalan dose (continuous variable) was significantly associated with severe neutropenia during cycles 2 and cycle 3 (p<0.0001, p<0.0001 by t-test, respectively). The minimum p-value approach showed that 0.50 mg/kg was the best cut-off point during cycle 2 (>0.50 versus ≤50 mg/kg, χ^2^ = 18.55, adjusted p = 0.0007) and 0.40 mg/kg during cycle 3 (>0.40 versus ≤0.40 mg/kg, χ^2^ = 26.26, adjusted p<0.0001). Melphalan dose>0.40 mg/kg was also associated with severe neutropenia during cycle 2 (χ^2^ = 12.34, adjusted p = 0.01).

### Analyses of treatment factors and severe neutropenia

#### Cycle 1

A univariate analysis regarding cycle 1 showed that a higher dose of melphalan (>0.40 versus ≤0.40 mg/kg) was significantly associated with severe neutropenia (p = 0.01, Table 2). The univariate analysis also showed that the severe neutropenia rate was significantly different among patients treated with different chemotherapy regimens (melphalan alone versus melphalan and either topotecan or carboplatin versus melphalan, topotecan and carboplatin, p = 0.04). Further pairwise comparison showed that patients treated with three chemotherapy agents more frequently experienced severe neutropenia compared to those treated with two agents (50% versus 9%, p = 0.02). However, a stepwise logistic regression revealed that while higher melphalan dose remained associated with severe neutropenia (odds ratio (OR) 4.11, 95% CI 1.33–12.73, p = 0.01), the number of chemotherapy agents administered did not.

**Table 2:** Cycle 1: Univariate analysis of severe neutropenia.

Variable	Severe neutropenia (25%)		p-value
	No (n = 55)	Yes (n = 18)	
M dose, mg/kg (mean ± SD)	0.39±0.14	0.43±0.12	
Median (range)	0.35 (0.15–0.89)	0.46 (0.27–0.68)	
≤0.40	37 (67%)	6 (33%)	0.01
>0.40	18 (33%)	12 (67%)	
Chemotherapy agents			0.04
M only	30 (55%)	11 (61%)	
M+ T/C	20 (36%)	2 (11%)	
M+ T + C	5 (9%)	5 (28%)	
Prior treatment^a^			0.77
No	24 (45%)	7 (41%)	
Yes	29 (55%)	10 (59%)	

M: melphalan; T: topotecan; C: carboplatin.

a Data not available for 3 patients (2 had and 1 did not have severe neutropenia).

#### Cycle 2

A univariate analysis revealed that a higher cycle 2 melphalan dose (>0.50 versus ≤0.50 mg/kg) was significantly associated with severe neutropenia (p<0.0001 by Chi-square test, adjusted p = 0.0007, Table 3). A stepwise logistic regression showed melphalan dose remained associated with severe neutropenia during cycle 2 (p = 0.0005, OR 10.86, 95% CI 2.84–41.57).

**Table 3:** Cycle 2: Univariate analysis of severe neutropenia^a^.

Variable	Severe neutropenia (30%)		p-value
	No (n = 45)	Yes (n = 19)	
M dose, mg/kg (mean ± SD)	0.36±0.12	0.51±0.13	
Median (range)	0.34 (0.04–0.57)	0.52 (0.26–0.71)	
≤0.50	40 (89)	7 (37)	<0.001^c^
>0.50	5 (11)	12 (63)	
≤0.40	31 (69%)	4 (21%)	0.01^c^
>0.40	14 (31%)	15 (79%)	
Chemotherapy agents			0.26
M only	22 (49%)	9 (47%)	
M+ T/C	20 (44%)	6 (32%)	
M+ T + C	3 (7%)	4 (21%)	
Prior treatment^b^			0.13
No	22 (51%)	5 (29%)	
Yes	21 (49%)	12 (71%)	
Cycle 1 grade 3 or 4 neutropenia			0.66
No	27 (60%)	9 (47%)	
No record	10 (22%)	5 (26%)	
Yes	8 (18%)	5 (26%)	

M: melphalan; T: topotecan; C: carboplatin.

a Only one variable was associated with severe neutropenia at p≤0.05 on univariate analysis; multivariate analysis was not performed.

b Data not available for 4 patients (2 had and 2 did not have severe neutropenia).

c Cut-off point selection adjusted p-value.

#### Cycle 3

A univariate analysis demonstrated that a higher cycle 3 melphalan dose (>0.40 versus ≤0.40 mg/kg) was once again significantly associated with severe neutropenia (p<0.0001, adjusted p<0.0001, Table 4). The univariate analysis and further pairwise comparison also showed that patients who had severe neutropenia during cycle 1 or 2 more frequently experienced severe neutropenia during cycle 3 compared to those who did not (yes versus unknown versus no, p = 0.02; yes versus no, p = 0.01). A stepwise multivariate logistic regression analysis revealed that while the higher melphalan dose remained associated with severe neutropenia (p = 0.0001, OR 72.0, 95% CI 7.93–653.4), severe neutropenia during cycle 1 or 2 did not.

**Table 4:** Cycle 3: Univariate analysis of severe neutropenia.

Variable	Severe neutropenia (34%)		p-value
	No (n = 33)	Yes (n = 17)	
M dose, mg/kg (mean ± SD)	0.34±0.11	0.50±0.08	
Median (range)	0.33 (0.13–0.70)	0.50 (0.33–0.69)	
≤0.40	27 (82%)	1 (6%)	<0.001^b^
>0.40	6 (18%)	16 (94%)	
Chemotherapy agents			0.15
M only	13 (39%)	4 (24%)	
M+ T/C	17 (52%)	8 (47%)	
M+ T + C	3 (9%)	5 (29%)	
Prior treatment^a^			0.83
No	16 (50%)	7 (47%)	
Yes	16 (50%)	8 (53%)	
Cycle 1 or 2, grade 3 or 4 neutropenia			0.02
No	14 (42%)	3 (18%)	
No record	11 (33%)	3 (18%)	
Yes	8 (24%)	11 (65%)	

M: melphalan; T: topotecan; C: carboplatin.

a Data not available for 3 patients (1 had and 2 did not have severe neutropenia).

b Cut-off point selection adjusted p-value.

### Impact of inevaluable cycles

A greater proportion of later cycles were inevaluable (31%, 36%, and 42% in cycles 1, 2, and 3, respectively) and that could introduce bias on generalization. However, there was no significant difference in melphalan dose (continuous or binary>0.40 versus ≤0.40 mg/kg), chemotherapy regimen, prior treatment, and severe neutropenia during cycle 1, 2 or 3 between the patients who had or had no record (at cycle 1, 2, or 3, p = 0.11 to 0.95), except that patients with record received a higher mean melphalan dose during cycle 1 (0.40±0.14 versus 0.34±0.14, p = 0.04). In addition, patients with record received a higher binary melphalan dose (>0.50 versus ≤0.50 mg/kg) during cycle 2 (27% versus vs 8%, p = 0.03).

### Clinical consequences of severe neutropenia

Most patients who developed severe neutropenia were asymptomatic. Two patients were admitted to the hospital during three periods of severe neutropenia due to fever and/or mucositis. Both patients made complete recoveries.

### Other severe hematological toxicities

Severe (grade 3 or 4) anemia or thrombocytopenia were encountered infrequently. Five patients suffered 1 episode each (5 episodes total) of grade 3 anemia and 2 patients suffered 1 episode each (2 episodes total) of grade 4 thrombocytopenia.

## Discussion

Intra-arterial chemotherapy was first introduced into the treatment regimen for patients with intra-ocular retinoblastoma by Reese and colleagues in 1955 when they administered triethylenemelamine via the internal carotid artery [2]. It then fell out of favor due to toxicity concerns (that included death) and lack of clear benefit over other treatments until 1987 when Kaneko and colleagues began to administer selective ophthalmic arterial injection of melphalan using a balloon catheter. Their group recently reported a retrospective series of 343 patients (408 eyes) treated with 1452 procedures from 1988 to 2007 [5]. They generally used single-agent melphalan at a dose of 5 to 7.5 mg/m^2^ and did not encounter any> grade 1 decrease of white blood cells. Using a 30∶1 conversion factor, these doses are approximately 0.17 to 0.25 mg/kg of melphalan.

In the patients reported in this series, we used a higher dose of melphalan (median dose of evaluable cycles 0.36 mg/kg, range 0.04 to 0.89 mg/kg) and have encountered severe neutropenia in 29% of the cycles. Fortunately, most of the episodes of severe neutropenia were grade 3 (n = 43) rather than grade 4 (n = 11) and were generally clinically insignificant. We counseled the parents about the risk of fever and neutropenia, but did not routinely prescribe filgrastim. We did not encounter any severe neutropenia during cycles in which the melphalan dose was ≤0.25 mg/kg (the approximate dose used in Japan). A limitation is that we may have overestimated the risk of neutropenia due to our decision to consider cycles with blood counts performed outside of the 7 to 14 day window without neutropenia to be inevaluable, but to consider cycles evaluable if grade 3 or 4 neutropenia was documented earlier or later in the cycle. We felt that potentially overestimating rather than underestimating the risk of neutropenia associated with the intra-arterial chemotherapy was the more conservative approach.

We hypothesized that (1) adding topotecan and/or carboplatin to the melphalan regimen and (2) a history of prior patient exposure to intravenous chemotherapy might be associated with increased risk for severe neutropenia, but that turned out not to be the case. The only factor that remained significant in analyses was melphalan dose, particularly when a dose of greater than 0.40 mg/kg was administered. Due to the relatively small number of events and sample size, we may not have enough power to detect some difference. We were also unable to select an optimal cut-off point of melphalan dose in a multivariate setting. Further study with a larger sample size may be warranted. This increased risk of neutropenia associated with a melphalan dose of greater than 0.40 mg/kg is comparable to the results in a small series reported by Argentine investigators. They noted that children receiving more than 0.48 mg/kg for bilateral tandem infusions had a significantly higher systemic area under the curve and a 50% probability of grade 3 or 4 neutropenia [6].

These data may help assist in the selection of safe intra-arterial chemotherapy doses for patients with intra-ocular retinoblastoma, but it is important to note that most patients who developed severe neutropenia were asymptomatic and did not require hospital admission for fever and neutropenia or an infection. To minimize the risk of severe neutropenia patients should be treated with a melphalan dose of 0.40 mg/kg or less, but should higher doses be required to adequately treat the retinoblastoma and cure the eye, the risk of severe neutropenia may be acceptable.
